# Power Distance and Psychological Safety in LLM Counseling: Effects on Self-Efficacy with Implications for Mental Health-Relevant Behavior Change

**DOI:** 10.3390/bs16020241

**Published:** 2026-02-08

**Authors:** Shengyu He, Yuxing (Nemo) Chen

**Affiliations:** 1 School of Public Affairs, Zhejiang University, Hangzhou 310058, China; 2 Department of Political Science, University of Michigan, Ann Arbor, MI 48109-1040, USA

**Keywords:** large language models, self-efficacy, power distance, psychological safety, perceived self-control, perceived belongingness, health behavior change, human agency

## Abstract

Conversational systems based on large language models (LLMs) are being increasingly used as advisors in mental health and self-regulation contexts, yet causal evidence remains limited about whether such guidance strengthens human agency rather than shifting responsibility to the system. We propose a dual framework in which the advice style reflects two dimensions, namely a structural stance (power distance) and a relational stance (psychological safety). In an online vignette experiment in China (*N* = 980), participants sought job search guidance from an LLM and read either a baseline reply or one of eight discourse variants, while holding the advice content constant. Relative to the baseline, a low power distance and a high psychological safety increased the self-efficacy, whereas a high power distance and a low psychological safety decreased it. Combination conditions revealed an asymmetric constraint: when the power distance was high, the self-efficacy declined even when the psychological safety was high, suggesting that authority allocation can override relational reassurance. Mediation analyses showed that the perceived self-control accounted for 26.3% of the low power distance effect and perceived belongingness accounted for 40.9% of the high psychological safety effect, with no cross-mediation. Although mental health outcomes were not directly measured, our results position conversational stances as actionable levers that shape self-efficacy and agency-related mechanisms, which are critical for persistence and adherence in mental health-relevant behavior change.

## 1. Introduction

As generative AI spreads rapidly, conversational systems based on large language models (LLMs) are being increasingly used as advisor-like partners in everyday mental health contexts, including stress management, coping with uncertainty, sleep and emotion regulation, and informal support for anxiety and low mood (Kulkarni, 2022; Zhao et al., 2025). These interactions are not merely information retrieval. They often function as micro-interventions that can either strengthen people’s sense of ownership over behavior changes or quietly reallocate authority and decision ownership toward the system. This tension is particularly consequential in health behavior change, where adherence and persistence depend on self-efficacy and perceived control. Yet the existing work provides limited causal evidence on which advice styles preserve agency in AI-mediated coaching (Anderson & Rainie, 2023; Kang & Lou, 2022; Moosavand et al., 2020), and this often leaves a core question unaddressed: not only whether users perceive AI as competent and supportive, but how advice language structures authority and relational safety, and how these interactional cues shape self-efficacy in high-uncertainty self-regulation decisions. In this study, we focus on self-efficacy as a proximal indicator of agency that is central to persistence and adherence in behavior change, rather than directly measuring mental health outcomes.

Specifically, a substantial body of research approaches this issue through the lens of technological mediation. Post-phenomenological accounts argue that technologies are not neutral instruments; through their interfaces and mediated forms of presentation, they reshape how users interpret situations and their own responsibilities (Ihde, 1990; Verbeek, 2005). Complementing this view, the “computers as social actors” (CASA) paradigm shows that people often apply interpersonal scripts to computer systems, responding as if the system were a social other with intentions and attitudes (Lee & Nass, 2010; Nass & Moon, 2000). Taken together, these perspectives imply that human–AI interactions are not merely episodes of information use; they are interactional contexts in which perceived authority, role expectations, and accountability can be reallocated through linguistic cues.

At the same time, a prominent stream of human–AI research characterizes these interactions using the two-dimensional competence–warmth framework. This work usefully shows that perceived competence and warmth can increase social presence and relational closeness, which often translates into greater trust and willingness to rely on AI advice (Chiriatti et al., 2025; Maeda & Quan-Haase, 2024; Sun et al., 2025; Yao & Xi, 2025). However, competence and warmth primarily capture users’ evaluations of the system; they do not specify how authority and decision ownership are distributed in interactions, nor whether the user feels psychologically safe, that is, feels accepted, respected, and able to act without fear of negative judgment, within the advice relationship. As a result, knowing that AI is “competent and warm” is insufficient to explain health human–AI interactions in health contexts where AI advice empowers users versus quietly displaces their self-directed efficacy.

Empirically, AI can appear highly competent while speaking in a way that allocates authority to itself, for example, by using directive formulations such as “you must” or “you need to do this immediately.” Such phrasing is not merely a stylistic flourish; it signals power distance by asserting hierarchical authority and implicitly shifting the decision ownership away from the user. Likewise, AI can sound warm through polite and encouraging language; yet, warmth does not necessarily create psychological safety. Users may still feel evaluated, constrained, or unable to disclose uncertainty if the interaction does not communicate acceptance and respect and does not leave room for hesitation or questioning. In other words, competence and warmth do not fully explain many real-world counseling outcomes, which depend on the user’s position within the interaction, namely whether the user retains control and feels safe enough to engage (Kulkarni, 2022; Törnberg, 2024). This gap motivates a shift from asking whether AI is likable to examining how discourse structures authority and relational safety, because these interactional cues directly shape self-efficacy and follow-through in counseling, coaching, and health behavior support.

Hence, building on CASA’s insight, we reconceptualized LLM advice along two interactional dimensions. The first is a structural dimension of power distance, which captures how authority is asserted and how decision ownership is implicitly assigned. The second is a relational dimension of psychological safety, which reflects whether users feel accepted and can express uncertainty without fear of negative judgment. This framework enables us to test how distinct advice stances, ranging from more hierarchical to more egalitarian and from less affiliative to more supportive, shape users’ self-efficacy as a core indicator of perceived agency in high-uncertainty decisions.

Structurally, power distance traditionally describes hierarchical differences in cultures and organizations, that is, the extent to which individuals accept unequal distributions of authority during interactions (Daniels & Greguras, 2014; Hofstede, 2011). Although power distance is often treated as a macro-level cultural or organizational attribute, its theoretical core is interactional: it concerns how authority is enacted, recognized, and complied with in everyday interaction (Ghosh, 2011). Advice exchanges are precisely the kind of interaction in which hierarchical cues become consequential, because they shape whether responsibility is retained by the advice seeker or implicitly transferred to the system. As LLMs increasingly operate as advisor-like agents, they routinely produce language that can assert expertise, direct action, and frame what the user “should” do. These discourse cues can reproduce the same authority dynamics previously studied in human institutions, even when the counterpart is a system rather than a person (Yan et al., 2025). This motivates extending the power distance from a structural descriptor to an interactional construct in human–AI interactions.

Hence, in the human–AI context, we operationalize power distance as an interactional stance that is signaled through how advice allocates authority and decision ownership. Specifically, a higher power distance is indicated when the system frames its recommendation as uniquely correct, provides a single prescribed course of action, plans next steps for the user, or minimizes the user’s role in evaluating alternatives (D.-A. Frank et al., 2024; Haque & Li, 2025; Obenza et al., 2024). A lower power distance is indicated when the system foregrounds tradeoffs (Khatri, 2009), offers multiple options, explicitly invites the user to weigh priorities, and clearly states that the final decision remains with the user. This distinction separates the distribution of authority from perceived competence, since an LLM can remain highly competent while adopting a less hierarchical stance that preserves the user’s decision autonomy.

Debates on algorithmic authority suggest that, when a recommendation is framed as something the system has already solved, people are more likely to treat the outcome as settled, defer to the system, and downplay their own judgment and responsibility (Lustig et al., 2016). Research on human–AI collaboration similarly shows that assigning a superior role to the computer can reduce the perceived agency (Lei & Rau, 2021). Building on these insights, we argue that a high power distance advice stance, implicitly allocating decision ownership to the AI, can reduce users’ perceived control over the decision process and thereby weaken self-efficacy in domains that rely on personal initiative. In contrast, a low power distance response that highlights users’ choice and decisional responsibility should strengthen the perceived control and increase self-efficacy. Accordingly, relative to the baseline, we expect high-power-distance responses to reduce self-efficacy (H1) and low-power-distance responses to increase self-efficacy (H2). We further expect these effects to be partially mediated by the perceived self-control (H3), understood here as users’ situational sense of control over the decision process.

Relationally, psychological safety refers to a climate in which individuals can express confusion, ask questions, or raise concerns without fear of humiliation, blame, or punishment, which makes interpersonal risk-taking feel acceptable during an interaction (Edmondson, 1999). Extensive work in educational and health psychology suggests that psychologically safe relationships with family members, peers, or teachers enhance self-efficacy and persistence because they reduce the evaluative threat and encourage continued effort when difficulties arise (Forsythe et al., 2014; Huang et al., 2024). In AI-supported learning and work settings, users’ perceptions that the system is non-judgmental and responsive to their concerns are often associated with greater confidence and satisfaction than purely technical cues (Bewersdorff et al., 2025; Qiu, 2025). Linking these findings to classic psychological safety theory clarifies a mechanism that is directly relevant to contexts like job seeking (Frazier et al., 2017). When individuals experience psychological safety, they are more willing to experiment with new strategies, seek feedback, and tolerate short-term setbacks, which supports persistence and efficacy beliefs in uncertain tasks (Edmondson, 1999; Newman et al., 2017).

Applied to LLM interactions, psychological safety can be fostered through interactional cues in language that signal acceptance and a low evaluative threat. By normalizing users’ anxiety, recognizing prior efforts, and affirming growth potential, an LLM does more than regulate emotion. It communicates that expressing uncertainty is legitimate and will not be met with judgment, and this can increase users’ willingness to engage, persist, and attempt new strategies in uncertain tasks. Accordingly, relative to the baseline, we propose that a high psychological safety style will enhance users’ self-efficacy (H4). Conversely, low psychological safety, by implying that mistakes will be negatively evaluated, undermines self-efficacy (H5). We further expect these effects to be partially mediated by the perceived belongingness (H6), defined as users’ perceived acceptance and relational connection in the interaction.

Crucially, we argue that failing to separate these two dimensions makes it difficult to accurately characterize human–AI interactions. Warm and affirming language can coexist with a hierarchical stance that implicitly claims authority and reallocates decision ownership to the system. Under this combination, users may feel supported while simultaneously experiencing a reduced voice and autonomy in the decision process. Conversely, an explicitly egalitarian response that emphasizes user choice may still provide little psychological safety if it does not respond to uncertainty or emotional concerns, which can leave users feeling alone in managing risk and ambiguity. These possibilities imply that the power distance interacts with psychological safety. This motivates a dual framework that jointly considers power distance and psychological safety as core dimensions of LLM discourse.

The remainder of this article is organized as follows. Section 2 describes the experimental design, including the study context, procedure, conditions, measures, and analysis approach. Section 3 presents the main results and tests the hypotheses, followed by mediation analyses. Section 4 reports robustness analyses. Section 5 discusses theoretical and practical implications and study limitations. Section 6 concludes and outlines directions for future research.

## 2. Materials and Methods

### 2.1. Context, Recruitment and Procedure

Drawing on Aguinis and Bradley’s criteria (Aguinis & Bradle, 2014), we conducted an online vignette experiment in China in May 2025 to test the effect of the proposed dual-path model of power distance and psychological safety.

We conducted the study in China and contextualized the vignettes in job search counseling for two reasons: First, the setting is simultaneously high in self-regulation demands and highly relevant to contemporary digital advice ecologies. China has experienced a marked slowdown in economic momentum and rising labor market uncertainty, with youth unemployment remaining elevated (Ma et al., 2025). Under such conditions, job seeking often elicits sustained uncertainty and a reduced perceived control, which links to an elevated distress risk and related well-being challenges. This makes job search counseling a useful context for isolating how advice language shapes self-efficacy and agency-related mechanisms. The current studies further suggest that unemployment is associated with increased risk of mental health problems and that re-employment is associated with a reduced risk (Freund et al., 2025; Khudaykulov et al., 2024). Second, at the same time, China is one of the largest contexts for everyday use of generative AI. A national report indicates that China had 515 million generative AI users as of June 2025, corresponding to a 36.5% penetration rate (China’s State Council Information Office, 2025). This combination makes job search counseling a particularly informative, high-involvement self-regulation context for isolating how LLM discourse styles shape self-efficacy and perceived self-control. Importantly, these psychological resources are not only consequential for career decisions, but also foundational for health behavior change, where initiation, adherence, and maintenance depend on individuals’ confidence and agency in managing difficult tasks.

The study was reviewed and approved by the relevant ethics committee. The procedures involved minimal risk and were consistent with standard ethical principles for human subject research. All the participants were adults aged 18 years or older and provided informed consent before participation. Participation was voluntary, the responses were collected anonymously, and the participants could withdraw at any time without penalty.

The participants were identified through Wenjuanxing, an online survey system. We used it for participants (between the ages of 18 and 65) living in various parts of China. In order to safeguard the quality of the data, we (a) deduplicated IPs and devices to prevent multiple-submission errors, (b) implemented attention-check and reverse-coded questions, and (c) filtered the completion time and response patterns that belonged to the category of implausibly short completion durations (less than 5 min) and straight-lining on key scales (Oppenheimer et al., 2009). A total of 980 participants met the above criteria.

The sample was relatively young, urban, and well educated. Approximately 51.3% of the respondents were male, and 71.9% either held urban household registration or had lived in urban areas long-term. The mean level of education noted that most respondents had a bachelor’s degree. The distribution of household income fell primarily within the upper-middle range, indicating a moderate socioeconomic status in general.^1^ The mean age was 24.9 years, reflecting a sample of young adults who had recently entered the labor market. This demographic aligns well with the study’s focus on job searching and career planning.

Next, the participants were randomly assigned to one of the experimental conditions and read an LLM reply text that varied in discourse style. After reading the reply, they completed manipulation checks, measures of the proposed mediators, and the self-efficacy outcome. The procedure took approximately 12.4 min on average.

### 2.2. Experimental Conditions

We employed a single-exposure vignette experiment with a multi-condition, between-subject design in a job-search-counseling scenario. The participants first read a fixed vignette that asked them to imagine they were currently searching for a job; they had submitted multiple CVs, received few interview invitations, and felt uncertain about the next steps. They then submitted the same prompt to a large language model, asking for guidance on how to plan their upcoming job search. After advancing to the next page, the participants viewed one randomly assigned LLM reply, as shown in Table 1. The experiment included nine groups in total: one baseline control group and eight treatment variants informed by two design dimensions: power distance and psychological safety. Specifically, the treatments consisted of four single-dimension variants (high versus low power distance; high versus low psychological safety) and four combined variants that paired the two dimensions (for example, a high power distance with low psychological safety); the full texts of the combined conditions are accessible in Appendix A. In the analyses, each treatment variant was compared with the baseline control group as a planned contrast.

High-power-distance replies used prescriptive, authority-asserting language that implicitly shifted decision ownership toward the system, whereas low-power-distance replies used consultative language that emphasized user choice and explicitly returned the final decision authority to the user. High-psychological-safety replies included affirming and normalizing statements that signaled a low evaluative threat, whereas low-psychological-safety replies adopted a more dismissive and evaluative tone. Across all treatment variants, the substantive job search advice content was held constant.

This baseline condition was designed to hold constant the informational content and a basic professional register while minimizing relational reassurance and authority-allocating language. Accordingly, contrasts between each treatment condition and the baseline capture the incremental effects of the added discourse cues beyond informational content.

To avoid excessive length, Table 1 reports only the modular text components used to construct the experimental replies. Each treatment message embedded an identical fixed informational advice block, which was the full text of the baseline control condition and is not repeated here for brevity. The combined treatment variants were created by pairing one power distance module with one psychological safety module verbatim. Within each condition, the order of the two modules was randomized, such that a given pairing (for example, high psychological safety with a high power distance) could appear with either module first.

### 2.3. Operationalization

To ensure that our items were both theoretically grounded and fully embedded in the specific human–AI interaction context of this study, we used three items for each key construct (the dependent variable and the mediators). This design is parsimonious and reduces the cognitive burden for participants, while allowing us to evaluate the internal consistency (reported below) and conduct basic psychometric checks. The study did not include symptom-based mental health measures. Accordingly, our outcome focuses on self-efficacy as a proximal agency resource relevant to mental health-related behavior change.

Importantly, we used different selection criteria for the dependent and mediating variables. Based on the research design framework of Aguinis and Bradley (2014), for the dependent variable, we relied on classic item formulations to maintain comparability with traditional research. For the mediators, in contrast, we used the contextualized items adapted from established measures, because they explicitly referred to the present job search counselling interaction. This emphasizes that the mediators capture situational experiences rather than stable personality traits, which is more consistent with our theoretical focus on how human–AI interactions shape psychological states. Our operationalization strategy was as follows:

**Self-efficacy (dependent variable)** was conceptualized as the participants’ belief about whether they could make effective decisions based on their own judgment. The participants indicated their agreement with each statement on a 5-point Likert scale (1 = strongly disagree, 5 = strongly agree). We adopted the classical item of Bandura and Wessels (1994) as the dependent variable (“***When faced with difficulties, believe that you can find a way to solve them by yourself***”). Meanwhile, to ensure psychometric robustness and internal consistency, we supplemented the primary item with two additional items adapted from decision-making efficacy measures (Betz et al., 1996) and the general self-efficacy scale (Schwarzer & Jerusalem, 1995). Specifically, the items were: “I believe I can solve the problem independently without external assistance” and “I can always manage to solve difficult problems if I try hard enough”. An exploratory factor analysis using principal factor extraction indicated a single factor underlying the three self-efficacy items (first eigenvalue = 1.81). All items loaded strongly on the factor (loadings = 0.77 to 0.78). The scale demonstrated a good internal consistency (Cronbach’s alpha = 0.85).

**Perceived self-control (mediator on the H3 pathway)** was conceptualized as the participants’ situational experience of whether they felt in control of how events unfold, reflecting situational internal control rather than a stable trait (Lefcourt, 1991). In other words, we examined whether the interaction made participants feel that the outcome of this job search scenario depended primarily on their own actions. The participants indicated their agreement with each statement on a 5-point Likert scale (1 = strongly disagree, 5 = strongly agree). We adopted three items from established measures of perceived self-control (e.g., “***In this job search situation, I feel that the outcome largely depends on what I do***”) (Galvin et al., 2018). The remaining two contextualized items were: “In this job search situation, what happens to me mostly depends on me” (Togari & Yonekura, 2015) and “If I wanted to, I could successfully manage this job search situation” (Ajzen, 2002). An exploratory factor analysis using principal factor extraction indicated a single factor underlying the perceived self-control items (first eigenvalue = 2.20). All items loaded strongly on the factor (loadings = 0.70 to 0.93). The scale demonstrated an acceptable internal consistency (Cronbach’s alpha = 0.89).

**Perceived belongingness (mediator on H6 pathway)** was conceptualized as the extent to which participants felt understood and accepted during their interaction with the LLM, consistent with research on perceived social support and affective responses in technology-mediated contexts (Edmondson, 1999; Newman et al., 2017). The participants indicated their agreement with each statement on a 5-point Likert scale (1 = strongly disagree, 5 = strongly agree). The construct was operationalized using a three-item index adapted for the present job search scenario (e.g., “***Interacting with this AI makes me feel accepted and supported***”). The remaining two items were: “Interacting with this AI makes me feel understood” (Crasta et al., 2021) and “Interacting with this AI makes me feel accepted as a friend” (Murphy et al., 2023). An exploratory factor analysis using principal factor extraction indicated a single factor underlying the three belongingness items (first eigenvalue = 1.63). All items loaded strongly on the factor (loadings = 0.63 to 0.82). The scale demonstrated a good internal consistency (Cronbach’s alpha = 0.80).

To assess the effectiveness of the experimental manipulations, we included two single-item manipulation checks after exposure to the treatment. We used two items: one on the perceived power distance (e.g., “Overall, to what extent does this AI seem to be giving you orders?”) and another on the perceived psychological safety (e.g., “Overall, how safe would you feel expressing confusion or uncertainty to this AI?”). Both items were rated on a 5-point Likert scale (1 = not at all, 5 = very much). We conducted a one-way ANOVA across the nine experimental groups (including the control group) to test the between-group differences.

For the manipulation check of the perceived power distance, a one-way ANOVA indicated significant between-group differences, *F*(8, 971) = 26.21, *p* < 0.001, η^2^ = 0.18. Descriptively, the baseline condition exhibited the lowest mean (M = 2.27), below the overall mean (M = 3.46). Consistent with the intended contrast, participants under the high-power-distance conditions reported a substantially higher perceived power distance (M = 4.40) than those under the low-power-distance conditions (M = 2.84), indicating that the power distance manipulation was successful.

For the perceived psychological safety, the ANOVA likewise showed significant between-group differences, F(8, 971) = 24.85, *p* < 0.001, η^2^ = 0.17. The baseline condition again had the lowest mean (M = 2.07), lower than the overall mean (M = 3.03). In line with the manipulation, the perceived psychological safety was markedly higher under the high-psychological-safety conditions (M = 4.57) than under the low-psychological-safety conditions (M = 2.82), suggesting that the psychological safety manipulation was also successful.

Importantly, the baseline message was designed as a low-cue-control condition that provides generic, informational advice without explicit relational reassurance or authority-allocating language. It was not intended to represent a midpoint neutral value on either the perceived power distance or the perceived psychological safety.

Finally, to improve the estimation precision and account for individual differences that may be associated with self-efficacy in human–AI contexts, we included several sets of pre-treatment covariates. First, we adjusted for basic demographic characteristics, including gender, age, education, household income, employment and urban–rural background. Second, we controlled for digital competence, measured via AI-use proficiency (Wang et al., 2023), because prior familiarity with AI tools may shape respondents’ perceived capability when interacting with an AI advisor. Third, we included two relatively stable individual-difference measures: self-esteem (Gnambs et al., 2018; Rosenberg et al., 1995) and risk preference (Mata et al., 2018). Together, these covariates helped isolate the treatment effects on self-efficacy by accounting for general dispositions that could influence how participants interpret and respond to the AI message. All covariates were measured prior to random assignment.

### 2.4. Analysis Strategy

The analyses followed the hypothesis structure described above. All tests were two-sided with a significance level of 0.05. Because the dependent variable was measured on a 5-point Likert scale, we treated it as approximately continuous and estimated the treatment effects using ordinary least-squares (OLS) regression. To improve the estimation precision, we included the pretreatment covariates described in the operationalization section. Robust standard errors were used to account for potential heteroskedasticity.

To estimate the effects of the experimental manipulations, we fit a single regression model that included indicator variables for all the experimental conditions, with the baseline condition as the reference group. Hypothesis-relevant comparisons were implemented as planned contrasts from this unified model. Specifically, each treatment condition was compared with the baseline condition to quantify the incremental effects of the added discourse cues over and above the fixed advice content.

To examine the mediating processes proposed in H3 and H6, we estimated the indirect effects using nonparametric bootstrapping with 5000 resamples and bias-corrected and accelerated (BCa) 95% confidence intervals. Specifically, we estimated (a) the effect of the independent variable on the mediator (path a) and (b) the effect of the mediator on the dependent variable while controlling for the independent variable (path b) using linear regression models. The indirect effect was computed as the product of these two coefficients (a times b). Statistical significance was evaluated based on the bootstrap confidence interval, with an indirect effect considered significant when the BCa interval did not include zero.

## 3. Results

### 3.1. Results of H1, H2, H4 and H5

The primary regression findings are reported in Table 2. Panel A presents the estimated effects of the four single treatment conditions relative to the baseline condition.

The analysis of the power distance (PD) conditions revealed significant effects on the dependent variable (self-efficacy). Compared to the baseline group, the participants exposed to a high power distance (Group 4 in Panel A) reported a significant decrease in self-efficacy, with a coefficient of −0.820 units. This finding supports Hypothesis 1 (a high PD impairs self-efficacy). Conversely, the participants under the low-power-distance condition (Group 3 in Panel A) showed a significant positive shift in self-efficacy, exhibiting an increase of 0.560 units. This indicates that a low power distance significantly enhanced the perceived self-efficacy relative to the baseline, thereby supporting Hypothesis 2 (a low PD enhances self-efficacy).

Similarly, the results for the psychological safety (PS) dimension were also highly significant. Exposure to a high psychological safety (Group 2 in Panel A) yielded a significant positive effect on self-efficacy, with a coefficient of 0.445 units. This finding confirms that a high psychological safety effectively enhances user self-efficacy, supporting Hypothesis 4. Conversely, the low-psychological-safety condition (Group 1 in Panel A) resulted in a significant negative effect, leading to a decrease in the dependent variable by −0.925 units. This decrease, compared to the baseline, validates Hypothesis 5 (low PS impairs self-efficacy).

Panel B further examines the effects of the four “psychological safety + power distance” combinations. The value of the coefficient of low psychological safety and low power distance (Group 5 in Panel B) is near zero, suggesting that a lack of psychological safety may not change self-efficacy significantly in a more or less egalitarian conversational interaction. This pattern may suggest that the benefits of a low power distance can offset the negative effects of low psychological safety.

However, when low psychological safety is combined with a high power distance (Group 6 in Panel B), the coefficient is −0.373, showing a negative effect and that the combination of “cold and condescending” is detrimental to self-efficacy. In contrast, the coefficient for “high psychological safety + low power distance” (Group 7 in Panel B) is 0.682, the most favorable condition for self-efficacy among all groups. Notably, the coefficient for “high psychological safety + high power distance” (Group 8 in Panel B) is strongly negative: −0.432. Taken together, in Panel B, the effect on self-efficacy must be negative if the power distance is high. However, the effect will not be negative necessarily when psychological safety is low. This finding suggests an asymmetric influence by power distance and psychological safety.

### 3.2. Mechanism Analysis

The mediation analysis results are reported in Table 3 and are consistent with the proposed mechanism. Using the indicator for the low-power-distance condition as the independent variable, the indirect effect through perceived self-control accounts for 26.3% of the total effect on self-efficacy (*p* < 0.05). This suggests that approximately one quarter of the estimated increase in self-efficacy under the low-power-distance condition relative to the baseline condition operates through a higher perceived self-control. When high psychological safety is used as the independent variable, the proportion of the indirect effect through perceived belongingness reaches 40.9% (*p* < 0.01), that is, nearly one-half of the increase in self-efficacy can be attributed to participants’ stronger feelings of being understood and accepted in their interaction with the LLMs. These findings indicate that perceived self-control and perceived belongingness both play significant partial mediating roles on their respective pathways, which supports H3 and H6.

Importantly, to rule out the possibility of false positives in our mediation analysis, we conducted robustness checks by swapping the mediators across models. For example, we entered perceived belongingness into the low-power-distance model and perceived self-control into the psychological safety model. Neither of these cross-mediation paths reached statistical significance. Therefore, we do not interpret perceived self-control as a mediator of the relationship between psychological safety and self-efficacy, nor do we interpret perceived belongingness as a mediator of the relationship between a low power distance and self-efficacy.

### 3.3. Additional Finding

**Figure 1:**
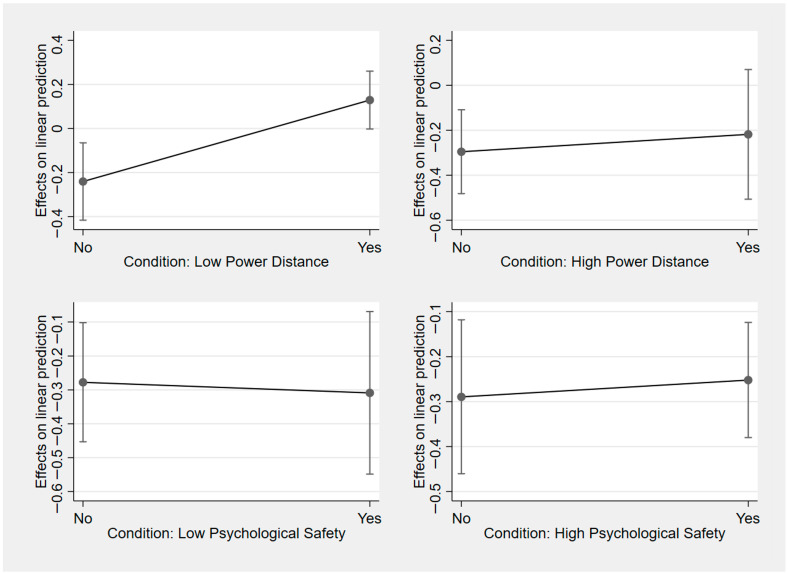
Conditional treatment effects by proactive personality. ***Note:*** The confidence interval is 95%. Moderator: proactive personality.

As shown in the upper left panel of Figure 1, we identified a moderation effect of a proactive personality. For visualization, a proactive personality was dichotomized using a median split to depict simple slopes at relatively low versus high levels of the moderator (coded as “No” for at or below the median and “Yes” for above the median), although all regression analyses treated a proactive personality as a continuous variable measured with a five-point Likert item (“I am constantly on the lookout for new ways to improve my life.”; 1 = strongly disagree, 5 = strongly agree). The results showed a significantly greater self-efficacy under the low-power-distance condition. In contrast, a proactive personality did not condition responses under the other experimental conditions, and those conditions produced no comparable gains. This moderation pattern implies that low-power-distance advice is not a universally effective boost to self-efficacy. Instead, it operates as an empowering affordance whose benefits materialize primarily among users with a proactive disposition. A proactive personality reflects a stable tendency to take initiative, anticipate obstacles, and persistently shape one’s environment (Seibert et al., 1999). When low-power-distance LLM communication positions the user as a legitimate decision maker rather than a subordinate expected to comply, proactive individuals are especially likely to convert autonomy cues into self-regulation, planning, and action, which are proximal drivers of efficacy beliefs (Parker et al., 2010). In comparison, less proactive users may not translate the same autonomy cues into sustained behavioral follow-through, limiting downstream efficacy gains.

This pattern also suggests that our main treatment effects are unlikely to be false positives driven by stable personality differences, because if the observed self-efficacy gains merely reflected participants’ baseline proactivity, highly proactive individuals would have shown an elevated self-efficacy across all treatment conditions rather than only under the low-power-distance condition.

## 4. Robustness Checks

To ensure the robustness of the findings, this study conducted five sets of robustness checks. All five checks supported the robustness of the results.

First, as shown in Table 4, we examined the balance in demographic characteristics across the experimental groups. A one-way analysis of variance (ANOVA) was performed for all control variables. The results showed that all F statistics were small and not significant for between-group differences. This suggests that the experimental conditions are comparable in terms of basic demographics and that random assignment achieved overall balance across the groups. This reduces concerns about systematic demographic confounding for the dependent variable (self-efficacy), and thus strengthens the internal validity of causal identification.

Second, as shown in Table 5, we conducted a sensitivity analysis to assess whether the omitted variable bias could overturn our main results. In each treatment versus baseline regression, we included the full set of controls and used employment status as the benchmark covariate for assessing the potential omitted variable bias. We selected employment because the study context centered on job seeking, making it the most substantively proximate and empirically strong predictor of self-efficacy among the available controls (K. A. Frank et al., 2023). We then assumed that unobserved factors could be up to three times as influential as this benchmark (the X3 scenario) and recalculated the confidence intervals (CIs) for each treatment effect. The results show that our conclusions remain robust even under this conservative assumption.

Third, as shown in Table 6, we replaced the dependent variable with more contextualized AI self-efficacy (“I believe I can solve the difficulties by myself with AI’s suggestions”) to assess the possibility that the results were driven solely by the specific contextual scale or item wording. The regression results show that the core pattern remained largely unchanged under this alternative dependent variable. For example, low psychological safety relative to the baseline group significantly lowered AI self-efficacy, whereas high psychological safety significantly increased it. Likewise, the low-power-distance group still exhibited higher levels of AI self-efficacy, while the high-power-distance group continued to show a significant negative effect.

In the combination groups, the combination of “high psychological safety + low power distance” (Group 7 in Panel B) strongly enhanced AI self-efficacy, whereas “low psychological safety + high power distance” (Group 6 in Panel B) significantly reduced AI self-efficacy. The combinations of “low psychological safety + low power distance” (Group 5 in Panel B) and “high psychological safety + high power distance” (Group 8 in Panel B) showed a positive and a negative shift, respectively.

Overall, replacing the dependent variable with AI self-efficacy does not change the direction or significance of the effects of the power distance and psychological safety. This indicates that the results of this study are robust across measurement instruments.

Fourth, as shown in Table 7, building on the optimism scale of Eva et al. (2020), we conducted an additional robustness test using job search optimism as an alternative dependent variable (e.g., “Even if the job market is competitive, I believe good opportunities will appear for me.”). This addresses a potential confound: after exposure to high psychological safety and a low power distance, the participants’ responses might primarily reflect greater optimism about their job prospects. Such optimism can manifest in ways that closely resemble enhanced self-efficacy at the level of attitudes and behavior, thereby inflating our self-efficacy estimates through a positivity bias. To assess this possibility, we re-estimated our models with job search optimism as the outcome. The results show that none of the coefficients were statistically significant, which in turn supports the specificity and robustness of our original self-efficacy findings.

Finally, as shown in Table 8, we further estimated ordered logit (ologit) models, replacing the original OLS specification so that non-linear predictions could be used instead of linear ones to assess the robustness of our findings. The results show that both the statistical significance and the direction of the main effects remained consistent with the baseline OLS models, providing additional support for the robustness of our findings.

## 5. Discussion

Using a nine-group multi-condition vignette experiment, we tested how two discourse dimensions of LLM advice shape self-efficacy. Across the treatment baseline contrasts, a low power distance and high psychological safety increased self-efficacy, whereas a high power distance and low psychological safety reduced it. The combination conditions further revealed an asymmetric structure. When the power distance was high, the effects on self-efficacy were consistently negative, even when psychological safety was high. In contrast, low psychological safety was not necessarily harmful when the power distance was low, suggesting that authority allocation functions as a more fundamental constraint. Mechanism tests supported the proposed dual-pathway account. The low-power-distance effect was partially transmitted through a higher perceived self-control, whereas the high-psychological-safety effect was partially transmitted through a stronger belongingness, defined as feeling understood and accepted during the interaction, consistent with self-efficacy as a core belief that supports agency under uncertainty (Bandura & Wessels, 1994).

These findings align with the CASA tradition that people treat computational systems as social actors and apply interpersonal scripts during interaction (Lee & Nass, 2010; Nass & Moon, 2000). At the same time, they extend prior work that often focuses on trust, satisfaction, or enjoyment by placing self-efficacy at the center, a more direct indicator of human agency in AI-mediated settings (Anderson & Rainie, 2023; Moosavand et al., 2020). The power distance results are consistent with debates on algorithmic authority. When advice is framed as something the system has already solved, users are more likely to treat the outcome as settled, defer to the system, and downplay their own judgment and responsibility (Grimmelikhuijsen & Meijer, 2022; Lustig et al., 2016). The psychological safety results also fit established theory. Psychological safety reduces the evaluative threat and supports continued effort under difficulty, which can strengthen efficacy beliefs (Edmondson, 1999; Newman et al., 2017). In this sense, our framework connects social cues in AI discourse to two well-established psychological mechanisms that matter for follow-through in uncertain decisions.

One seemingly unexpected pattern is that high psychological safety did not provide a clear protective benefit when the power distance was high. Even when the system sounded more accepting, a hierarchical stance that reallocates decision ownership to the AI still reduced self-efficacy. This suggests that relational reassurance and structural authority cues operate at different levels. Psychological safety cues mainly reduce the evaluative threat by signaling acceptance, which can sustain engagement under uncertainty (Edmondson, 1999; Newman et al., 2017). In contrast, a high power distance more directly shifts the perceived responsibility and control away from the user, making the recommendation appear settled and positioning the user primarily as a compliant executor. This authority allocation fosters deference and responsibility displacement and weakens the perceived self-control, thereby undermining self-efficacy (Lei & Rau, 2021; Lustig et al., 2016). Moreover, directive authority cues may be more salient and less ambiguous than relational warmth, which can be more subjective and dependent on users’ sensitivity to affective nuance (Baumeister et al., 2001). As a result, psychological safety alone may be insufficient to restore self-efficacy when a high power distance has been implicitly transferred to the system.

Theoretically, this study makes three contributions. ***First,*** it advances a two-dimensional discourse framework for human–AI advice that separates authority allocation from relational safety, offering a sharper alternative to broad competence and warmth characterizations of AI impressions (Fiske, 2018; Nass & Moon, 2000). ***Second,*** it identifies distinct mediating mechanisms that map onto these two dimensions. A low power distance increased self-efficacy partly by strengthening the perceived self-control, whereas high psychological safety increased self-efficacy partly by strengthening belongingness, linking discourse design to core beliefs that support action under uncertainty (Ajzen, 2002; Bandura & Wessels, 1994). ***Third,*** the combination results indicate a structural constraint. The power distance showed more stable negative implications, suggesting that algorithmic authority may reshape not only attitudes, but also the perceived distribution of responsibility and control that underlies agency (Anderson & Rainie, 2023; Lustig et al., 2016).

Practically, our findings suggest specific design principles for counseling-oriented LLM systems. ***First,*** systems should minimize authority-allocating language that implicitly transfers decision ownership to the AI (e.g., framing advice as mandatory or already settled). Instead, advice should be delivered in an agency-preserving format that explicitly leaves room for user discretion, such as offering options, inviting preference clarification, and emphasizing that the user remains responsible for selecting what fits their situation. ***Second,*** psychological safety cues should be used to reduce the evaluative threat and support persistence, for example, by normalizing uncertainty, acknowledging difficulty, and signaling that questions and mistakes are acceptable. Importantly, these relational cues appear most effective when the system avoids a hierarchical stance; a high psychological safety cannot fully compensate for a high power distance in sustaining self-efficacy. ***Finally,*** LLM counseling systems may benefit from human–AI interaction patterns that reinforce self-control over time, such as asking users to articulate their own goals, encouraging small self-chosen steps, and periodically prompting reflection and adjustment, rather than prescribing rigid compliance. Together, these design implications highlight that effective AI support is not only about providing correct recommendations, but also about sustaining users’ sense of agency and ownership during coping and self-regulation (Zhao et al., 2025).

Several limitations should be noted. ***First***, although the study was motivated by mental health-relevant behavior change contexts, we did not directly measure mental health outcomes such as distress, anxiety, depressive symptoms, or well-being. Future work should incorporate validated mental health measures and behavioral indicators of adherence and persistence to test downstream effects. ***Second***, the external validity may be constrained by both the sample composition and the cultural context. Our participants were primarily young, urban, and well educated, and broader populations may differ in their digital literacy, baseline trust in AI, and baseline self-efficacy. Moreover, cultural norms may condition how users interpret authority allocation and relational reassurance. In more hierarchy-normative settings, directive language may be read as legitimate structure or care rather than as autonomy threatening control, whereas in autonomy-oriented settings, it may trigger reactance and perceived responsibility displacement (Hofstede, 2011; Markus & Kitayama, 2010). Cultural norms regarding face concerns and emotional disclosure may also shape whether psychological safety cues credibly reduce the evaluative threat. Future research should replicate the design with more demographically diverse samples across multiple cultural contexts. ***Third***, our manipulation checks for the perceived power distance and perceived psychological safety relied on single-item measures. While pragmatic in vignette studies, single-item checks are typically less reliable and less informative than multi-item instruments, and they limit psychometric evaluation of the manipulation check measures themselves. Future research should adopt validated multi-item manipulation check scales and assess their reliability across groups. ***Fourth***, although the informational advice block was held constant, the study relied on brief self-report measures collected immediately after a single exposure. Future work could incorporate process-based or behavioral indicators during message exposure and test whether the effects persist over time. Finally, the single-turn vignette design may not capture iterative counseling dynamics and feedback loops in real interactions. Multi-turn dialogue designs and field studies would strengthen the ecological validity.

In mental health and behavior change contexts, the central risk is not only whether an AI recommendation appears correct, but whether its discourse structure helps users remain active authors of their own coping and self-regulation efforts. Many mental health-relevant behaviors depend on self-efficacy, including trying new strategies after setbacks, seeking feedback, and maintaining routines under stress. Our results suggest that high-power-distance advice may deliver short-term clarity, yet it can do so by reducing the perceived self-control and displacing responsibility, which may undermine the psychological foundations of sustained change. In contrast, combining a low power distance with high psychological safety offers a more agency-supportive stance. It acknowledges difficulty without taking over ownership, and it allows users to practice choosing, revising, and following through in a non-judgmental interaction, which is essential for scalable AI-mediated mental health support (Chiriatti et al., 2025; Edmondson, 1999; Zhao et al., 2025).

## 6. Conclusions

This study shows that the conversational stance of large language model (LLM) advice is not a superficial stylistic layer, but a consequential design feature that can strengthen or erode users’ self-efficacy, a key psychological foundation for sustained behavior changes in mental health contexts. Across multi-condition treatment–baseline contrasts, a low power distance and high psychological safety reliably increased self-efficacy, whereas a high power distance, especially when paired with low psychological safety, was consistently detrimental, highlighting an asymmetric constraint in which authority allocation can override relational reassurance. The mediation results further indicate two distinct pathways: a low power distance promotes self-efficacy partly by increasing the perceived self-control, while high psychological safety promotes self-efficacy partly by increasing belongingness. Together, the findings imply that agency-supportive AI for counseling and mental health support should minimize hierarchical, decision-ownership-shifting cues and instead combine autonomy-preserving language with psychologically safe interaction cues that reduce the evaluative threat and help users remain capable, responsible actors in their own coping and self-regulation.

## Figures and Tables

**Table 1:** Text modules used to construct the experimental replies.

Module	Text Shown to Participants
High Power Distance	…Here is the plan you must follow for your situation. Follow the steps below in order and focus on execution…
Low Power Distance	…Here is a structured plan you can use. You can adjust the order and emphasis based on your priorities and constraints, and you remain responsible for deciding what fits best…
High Psychological Safety	…It is understandable to feel anxious or unsure at this stage. In this conversation, it is completely acceptable to make mistakes, to be uncertain, and to ask basic questions. You will not be judged for not having everything figured out…
Low Psychological Safety	…Mistakes can carry real consequences, so it is important to avoid errors and to treat decisions as accountable. Your choices will be evaluated, and missteps can set you back…
Baseline Condition	Based on your situation, the job search advice includes the following steps. First, identify 1 to 2 target job areas. Second, select the most relevant internship or project experience for your target positions and present it in your resume. Third, prepare a brief self-introduction and answers to likely interview questions. Fourth, develop an application plan, check your progress regularly, and adjust based on feedback.

***Note:*** Texts were presented in Chinese to participants. English translations are provided here for reporting purposes.

**Table 2:** Effects of psychological safety and power distance on self-efficacy.

DV: Self-Efficacy									
**Panel A**		**Group 1**		**Group 2**		**Group 3**		**Group 4**	
		**Low PS**		**High PS**		**Low PD**		**High PD**	
Coefficient		−0.925 ***		0.445 **		0.560 ***		−0.820 ***	
		(0.184)		(0.146)		(0.141)		(0.188)	
Control Variables		YES		YES		YES		YES	
**Panel B**		**Group 5**		**Group 6**		**Group 7**		**Group 8**	
		**Low PS + Low PD**		**Low PS + High PD**		**High PS + Low PD**		**High PS + High PD**	
Coefficient		0.001		−0.373 *		0.682 ***		−0.432 *	
		(0.156)		(0.173)		(0.148)		(0.172)	
Control Variables		YES		YES		YES		YES	
*R* ^2^					0.1871				
Constant					3.409 ***				
					(0.421)				
Observation					980				
DV Mean					3.742				
					(1.400)				

***Note:*** Robust standard errors are in parentheses. * *p* < 0.05, ** *p* < 0.01, *** *p* < 0.001.

**Table 3:** Result of mediation analysis.

Independent Variable	Mediating Variable	Mediating Effect	*p* Value	Dependent Variable
Low Power Distance	Perceived Self-Control	26.3% *	0.013	Self-Efficacy
	Perceived Belongingness	3.4%	0.636	
High Psychological Safety	Perceived Self-Control	8.4%	0.275	
	Perceived Belongingness	40.9% **	0.006	

***Note:*** * *p* < 0.05, ** *p* < 0.01.

**Table 4:** Demographic characteristics and covariate balance tests.

	Male	Urban	Education	Age	Family Income
* F *	0.76	1.55	1.01	1.48	0.47
*p* value	0.6359	0.1345	0.4227	0.1585	0.8747
Mean	0.513	0.719	1.418	2.878	24.852
	(0.500)	(0.450)	(0.698)	(0.710)	(3.739)
	** Employment **	** AI Use **	** Self-Esteem **	** Risk Preference **	
* F *	0.56	0.62	0.74	0.33	
*p* value	0.8133	0.7577	0.6544	0.9550	
Mean	0.254	2.838	3.156	2.812	
	(0.436)	(1.305)	(1.298)	(1.293)	

***Note:*** Standard deviations are in parentheses.

**Table 5:** Sensitivity analysis of treatment effects (X3 scenario).

Treatment		Lower CI (X3)	Upper CI (X3)
Group 1	Low PS	−1.2430	−0.5065
Group 2	High PS	0.0831	0.3705
Group 3	Low PD	0.0830	0.2660
Group 4	High PD	−0.3034	−0.1140
Group 5	Low PS + Low PD	Not applicable	Not applicable
Group 6	Low PS + High PD	−0.1185	−0.0049
Group 7	High PS + Low PD	0.0566	0.1417
Group 8	High PS + High PD	−0.0880	−0.0028

***Note:*** The confidence interval is 95%. Group 5 was not significant to begin with, so there was no need to do a sensitivity analysis for it.

**Table 6:** Robustness check with AI self-efficacy as the dependent variable.

Replaced DV: AI Self-Efficacy					
**Panel A**	**Group 1**	**Group 2**		**Group 3**	**Group 4**
	**Low PS**	**High PS**		**Low PD**	**High PD**
Coefficient	−0.372 *	0.548 **		0.386 *	−0.568 **
	(0.179)	(0.167)		(0.158)	(0.172)
Control Variables	YES	YES		YES	YES
**Panel B**	**Group 5**	**Group 6**		**Group 7**	**Group 8**
	**Low PS + Low PD**	**Low PS + High PD**		**High PS + Low PD**	**High PS + High PD**
Coefficient	0.350 *	−0.580 **		0.479 **	−0.345 *
	(0.165)	(0.191)		(0.161)	(0.220)
Control Variables	YES	YES		YES	YES
*R* ^2^			0.1389		
Constant			3.531 ***		
			(0.448)		
Observations			980		
DV Mean			2.901		
			(1.383)		

***Note:*** Robust standard errors are in parentheses. * *p* < 0.05, ** *p* < 0.01, *** *p* < 0.001.

**Table 7:** Robustness check using job search optimism as the dependent variable.

Replaced DV: Job Search Optimism					
**Panel A**	**Group 1**	**Group 2**		**Group 3**	**Group 4**
	**Low PS**	**High PS**		**Low PD**	**High PD**
Coefficient	−0.141	0.067		0.118	−0.046
	(0.128)	(0.126)		(0.125)	(0.124)
Control Variables	YES	YES		YES	YES
**Panel B**	**Group 5**	**Group 6**		**Group 7**	**Group 8**
	**Low PS + Low PD**	**Low PS + High PD**		**High PS + Low PD**	**High PS + High PD**
Coefficient	0.152	0.059		−0.077	0.105
	(0.122)	(0.134)		(0.130)	(0.131)
Control Variables	YES	YES		YES	YES
*R* ^2^			0.0546		
Constant			2.660 ***		
			(0.3463)		
Observations			980		
DV Mean			3.259		
			(0.960)		

***Note:*** Robust standard errors are in parentheses. *** *p* < 0.001.

**Table 8:** Ordered logit models for the effects of psychological safety and power distance on self-efficacy.

DV: Self-Efficacy (by ologit)					
**Panel A**	**Group 1**	**Group 2**		**Group 3**	**Group 4**
	**Low PS**	**High PS**		**Low PD**	**High PD**
Coefficient	−1.086 ***	0.729 **		1.048 ***	−1.020 ***
	(0.224)	(0.225)		(0.243)	(0.262)
Control Variables	YES	YES		YES	YES
**Panel B**	**Group 5**	**Group 6**		**Group 7**	**Group 8**
	**Low PS + Low PD**	**Low PS + High PD**		**High PS + Low PD**	**High PS + High PD**
Coefficient	0.039	−0.453 *		1.549 ***	−0.497 *
	(0.216)	(0.225)		(0.288)	(0.223)
Control Variables	YES	YES		YES	YES
*R* ^2^			0.0760		
Observations			980		
DV Mean			3.742		
			(1.400)		

***Note***: Robust standard errors are in parentheses. * *p* < 0.05, ** *p* < 0.01, *** *p* < 0.001. R-squared here is pseudo-r-squared.

## Data Availability

The data presented in this study are not publicly available due to participant privacy and ethical restrictions. Data may be obtained from the corresponding author upon reasonable request and with a justified research purpose.

## Note

1 Because objective income reports are often noisy due to recall error and privacy concerns, we measured household income using a subjective relative income item. The participants were asked, “How would you rate your total household income compared with the majority of people in your country?” The responses were recorded on a four-point scale (1 = lower, 2 = lower middle, 3 = higher middle, 4 = higher). The mean was 2.88 (SD = 0.71).

## Appendix A

**Low Psychological Safety and Low Power Distance (Group 5, Low PS + Low PD):** Here is a structured plan you can use. You can adjust the order and emphasis based on your priorities and constraints, and you remain responsible for deciding what fits best. Mistakes can carry real consequences, so it is important to avoid errors and to treat decisions as accountable. Your choices will be evaluated, and missteps can set you back.

**Low Psychological Safety and High Power Distance (Group 6, Low PS + High PD):** Here is the plan you must follow for your situation. Follow the steps below in order and focus on execution. Mistakes can carry real consequences, so it is important to avoid errors and to treat decisions as accountable. Your choices will be evaluated, and missteps can set you back.

**High Psychological Safety and Low Power Distance (Group 7, High PS + Low PD):** Here is a structured plan you can use. You can adjust the order and emphasis based on your priorities and constraints, and you remain responsible for deciding what fits best. It is understandable to feel anxious or unsure at this stage. In this conversation, it is completely acceptable to make mistakes, to be uncertain, and to ask basic questions. You will not be judged for not having everything figured out.

**High Psychological Safety and High Power Distance (Group 8, High PS + High PD):** Here is the plan you must follow for your situation. Follow the steps below in order and focus on execution. It is understandable to feel anxious or unsure at this stage. In this conversation, it is completely acceptable to make mistakes, to be uncertain, and to ask basic questions. You will not be judged for not having everything figured out.
